# Structural features beneath neuronal avalanches

**DOI:** 10.1186/1471-2202-14-S1-O18

**Published:** 2013-07-08

**Authors:** Leonardo P Maia, Thiago S Mosqueiro

**Affiliations:** 1 Instituto de Física de São Carlos, Universidade de São Paulo, 13560-970, São Carlos, SP, Brazil

## 

Recently, Friedman *et al *performed high-resolution measurements [1] that strongly supported an universal critical character (in the sense of statistical physics) of neuronal avalanches, despite the abnormally high frequency of large events ("bumps" in the distributions of size and lifetime of avalanches) deforming the pure power-law behavior expected from analogies to equilibrium critical phenomena that became manifest only for smaller events.

Based on simulations of the Kinouchi-Copelli (KC) model, we have shown [2] that such bumps may not be experimental artifacts and really be typical at criticality when the topology of the neural network is the Barabási-Albert (BA) model, leading to a scale-free degree distribution of exponent -3.0. On the other hand, those simulations could not reproduce the exponents of the power-law region of the avalanche distributions in [1] (namely, -1.7 for the size distribution and -1.9 for the lifetime distribution). Besides that, the KC dynamics on BA topology revealed that the information capacity (entropy of avalanche size distribution) did not exhibit critical optimization, in contrast with an earlier experiment [3].

In this study we investigate the KC dynamics on the Uncorrelated Configuration Model (UCM) [4]. The UCM is a kind of "wiring procedure" of a neural topology that can lead to scale-free degree distributions with tunable exponents and can be "matched" to BA model. However, even so their avalanches are not identical. While the UCM also allows the appearance of bumps on the avalanche distributions, it both shows that the information capacity may be critically optimal and exhibits quantitatively accurate values of the critical exponents for small avalanches, suggesting the UCM may be descriptive of some structural features in the systems claimed to exhibit critical dynamics.
